# Production of valuable chemicals from glycerol using carbon fiber catalysts derived from ethylene

**DOI:** 10.1038/s41598-021-99210-2

**Published:** 2021-10-12

**Authors:** Anna Malaika, Karolina Ptaszyńska, Mieczysław Kozłowski

**Affiliations:** Faculty of Chemistry, Adam Mickiewicz University in Poznań, Uniwersytetu Poznańskiego 8, 61-614 Poznań, Poland

**Keywords:** Engineering, Materials science, Nanoscience and technology

## Abstract

Ethylene was thermocatalytically transformed into carbon products via a CCVD process. The filamentous carbon obtained was further modified with concentrated sulfuric acid or 4‐benzenediazonium sulfonate (BDS) to produce acid-type catalysts. The as-prepared samples were characterized by SEM and TEM techniques to confirm their morphological features. TG, XRD, elemental, and porosity analyses were also performed to assess the quality of these materials. The fabricated carbons were tested in eco-friendly green synthesis of value-added fuel bio-additives, namely in glycerol esterification. The reaction of glycerol transformation was performed with acetic acid at 80 °C using different glycerol to acetic acid (Gly/AA) molar ratios. The samples functionalized with diazonium salt showed better performance in the above process than those modified with H_2_SO_4_, and this was found to be directly related to the degree of surface functionalization with acidic sites. BDS-modified carbon fibers allowed obtaining acceptable results within 6 h when the reaction was performed with a Gly/AA molar ratio of 1:6, however, the dominant products in this case were mono- and diacetins. Extended reaction time altered the distribution of products. Finally, the combined selectivity to the targeted acetins (i.e., DA and TA) was about 75.5%. A direct correlation between the content of –SO_3_H groups of CNFs and the yield of higher acetins was found.

## Introduction

Glycerol has recently become an important chemical platform for obtaining valuable compounds such as acetins, solketal, dihydroxyacetone etc. applying sustainable strategies^1,2^. This is related to the large market supply of low-cost, non-petroleum derived glycerol (i.e., bio-glycerol), which is formed as a by-product of biodiesel synthesis, as well as to a number of possibilities to effectively convert glycerol into industrially important products^3–5^.

Esterification of glycerol using acetic acid presents a promising and economically viable approach to synthesize mono-, di- and triacetates (also called mono,- di- and triacetins—MA, DA and TA, respectively) of significant industrial importance. These products are applied in many areas, such as food, pharmaceutical, cosmetic, plastic, chemical, or fuel industries. For instance, MA serves as a plasticizer for cellulose-derived and vinylidene-based polymers, a solvent for dyes, and a reagent in the production of tanners and explosives^6,7^. Whereas, DA is used as a plasticizer, softening agent and a solvent^6^, a mixture of MA and DA is applied for the production of biodegradable polyesters as well as used as an emulsifier in pharmaceutical, food, and cosmetic industries^8^. Moreover, DA and TA are recognized to be excellent biofuel enhancers and good alternatives to conventionally used tertiary alkyl ethers such as methyl tert-butyl ether (MTBE) and ethyl tert-butyl ether (ETBE)^7,9^.

Traditionally, glycerol esterification is performed with strong Brönsted acids, such as H_2_SO_4_ or p-toluenesulfonic acid^10^. However, to make the process “greener” and more eco-friendly, the usage of heterogeneous acid catalysts has been suggested for this reaction, as these catalysts are easy to separate and recycle as well as less invasive and toxic than their homogeneous counterparts^10^. Several solid acid catalysts and reaction set-ups have been tested in glycerol acetylation. For instance, Zhou et al.^11^ performed acetylation of glycerol with acetic acid over ion exchange resin Amberlyst 15 using a slurry reactor. Gonçalves et al.^12^ compared the activity of different solid acids such as Amberlyst 15, K-10 montmorillonite, niobic acid, or zeolites in glycerol acetylation performed in a batch mode. Further, Balaraju et al.^13^ used niobic acid supported tungstophosphoric acid (TPA) catalysts with varying TPA content. Kulkarni et al.^14^ examined metal oxide CeO_2_–ZrO_2_ catalysts, and Nandiwale et al.^15^ tested H_2_SO_4_-modified K10 clay. As gathered in Table 1, some of these catalysts converted glycerol into acetins inefficiently, other achieved high glycerol conversions, but at the same time presented limited selectivity to the most valuable DA and TA. There were also catalysts that exhibited very high activity towards formation of di- and triacetins, however, these materials often required the use of high-elevated reaction temperature and a high acetic acid to glycerol molar ratio to reach reasonable catalytic results. It should also be stressed that commercially available Amberlyst 15 usually displayed significant activity in glycerol acetylation, but this catalyst is thermally unstable above 120 °C^16^. This could be a limiting factor for designing the industrial scale process.

**Table 1:** Comparison of catalytic performances of different catalysts in glycerol acetylation with acetic acid.

Sample	Gly/AA molar ratio	Temp. (°C)	Time (h)	Selectivity (%)			Glycerol conversion (%)	References
				MA	DA	TA		
SO_4_^2−^/CeO_2_-ZrO_2_	1:10	100	3	22	57	21	99	^14^
PT800S	1:6	120	3	~ 5.8	32.2	58.9	97.5	^10^
Amberlyst 15	1:6	80	6	23	59.5	17.5	~ 99	^18^
Amberlyst 15	1:9	110	5	nd	47.7	44.5	97	^11^
Niobic acid	1:3	nd	0.5	~ 83	0.0	0.0	~ 30	^12^
K-10 clay	1:3	nd	0.5	44	49	5	96	^12^
20%SO_4_/K10	1:12	120	5	23(*Y)	59(*Y)	15(*Y)	99	^15^
HZSM-5	1:3	nd	0.5	83	10	0.0	30	^12^
25%TPA/Nb_2_O_5_	1:5	120	4	~ 22	~ 58	20	~ 98	^13^
N-based Brønsted-acidic ionic liquids	1:10	90	0.5	4.8	52.9	42.3	99.3	^25^
Purolite CT-275	1:9	100	6	nd	~ 59(*Y)	~ 24(*Y)	100	^26^
OMSC	1:10.4	126	3	4.9	27.8	66.5	97	^27^
2 M SO_4_^2−^/γ-Al_2_O_3_	1:12	110	nd	22.8	49.8	27.4	99.2	^28^
TC-L	1:5	150	5	10	52	38	90	^21^
C_glycerol	1:6	110	2	21	56	23	97	^20^

*Y—yield.

*Gly* glycerol, *AA* acetic acid, *MA* monoacetins, *DA* diacetins, *TA* triacetin, *PT800S* sulfonated carbon from palm kernel shell biomass obtained with a template method, *OMSC *sulfonated mesoporous carbon derived from palm kernel shells, *TC-L* sulfonated carbon from rice husk, *C_glycerol* carbon obtained from glycerol by partial carbonization.

Carbon-based materials are a group of catalysts that are more and more often used in glycerol acetylation^10,17–22^. This is due to several reasons. First, carbons have a set of unique features, such as high surface area, resistance to acidic/basic media, high thermal stability, or controllable porosity and chemistry, which makes them perfect candidates for numerous applications^23,24^. Secondly, these materials are generally quite cheap and easily produced as well as are recognized as environmentally benign^24^. Finally, studies show that carbons can work perfectly in glycerol esterification, being a real alternative to conventional, expensive solid acid catalysts. For instance, Nda-Umar et al.^10^ tested sulfonated mesoporous carbons obtained from palm kernel shell biomass and found that under the optimized conditions, these samples are efficient in catalyzing glycerol esterification to higher acetins (i.e., DA and TA) within a short period of 3 h. Our team conducted studies with glycerol- and sugar-derived one-pot bio-carbons and demonstrated high conversion of glycerol and high selectivity to DA and TA achieved with these samples in just 2 h^20^. Further, Carvalho et al.^21^ reported good catalytic performance of carbons obtained from rice husk. Detailed results of these studies are presented in Table 1.

CNFs have been successfully used as catalysts, catalyst supports, electrodes for fuel cell devices, in nanocomposites, or in hydrogen storage systems^29,30^ for many years. In our previous work, we presented results of the quantitative studies regarding the formation of CNFs from ethylene^31^. The aim of the current research is presenting the possibility of application of these interesting carbon materials in glycerol acetylation leading to acetins. To the best of our knowledge, this is the first time when such kind of samples has been used in the reaction between glycerol and acetic acid.

## Experimental part

The conducted studies were aimed at production, characterization and application of filamentous carbon in glycerol acetylation. The sections below describe in detail the conditions of sample preparation, characterization as well as testing.

### Synthesis of carbon growth catalysts

Unsupported Ni and 10%Ni/CaCO_3_ were used as the growth catalysts for the CNF production.

In order to obtain the unsupported Ni catalyst, the following procedure was used. First, nickel(II) nitrate hexahydrate [Ni(NO_3_)_2_·6H_2_O, p.a. EMSURE ACS, Sigma Aldrich] was dissolved in distilled water and mixed with ammonium bicarbonate (NH_4_HCO_3_, p.a., Aktyn, Poland). The precipitate formed in the process was filtered and washed with distilled water, dried overnight at 120 °C, and sieved to a particle size of ≤ 0.4 mm. Finally, the resulting sample was subjected to calcination at 500 °C in air for 3 h (heating rate of 5 °C/min) to produce the corresponding metal oxide. Obtaining the pure metal catalyst required the reduction of the metal oxide sample, which was performed just before the CCVD process (as described below).

The supported Ni system was prepared by incipient wetness impregnation of a dry support, which was CaCO_3_ (p.a., Avantor Performance Materials, Poland), with a nickel(II) nitrate hexahydrate [Ni(NO_3_)_2_^.^6H_2_O, p.a. EMSURE ACS, Sigma Aldrich] solution at room temperature for 24 h. The obtained material was dried at 120 °C overnight, and finally calcined at 500 °C for 3 h (heating rate of 5 °C/min). The reduction of the oxide sample was performed with a H_2_/Ar mixture before the process of CNF growth (details presented below) to obtain metallic Ni (10wt.%) on CaCO_3_.

### Preparation of carbon nanofibers

Carbon nanofibers (CNFs) were produced using ethylene as a carbon feedstock, which was applied either alone (i.e., 100%C_2_H_4_) or combined with hydrogen (25%C_2_H_4_/75%H_2_). The synthesis of CNFs was carried out in a horizontal tube furnace at 550 °C. Before each run, a weighed sample of the growth catalyst (250 mg in the case of Ni oxide/CaCO_3_ and 30 mg for unsupported Ni oxide) was reduced at 550 °C with a mixture of hydrogen and argon (20%H_2_/80%Ar, flow rate of 100 cm^3^/min) for 2 h to obtain the supported or unsupported Ni catalyst. After this step, the carbon precursor gas (which was ethylene when 10%Ni/CaCO_3_ was used or C_2_H_4_/H_2_ mixture when pure Ni was applied) was passed through the CCVD reactor at the total flow rate of 100 cm^3^/min for 4 h. After completion of the reaction, the resulting carbon product was boiled with a 21% solution of HCl for 2 h. Subsequently, the obtained carbon structures were filtered, washed thoroughly with hot distilled water, and dried overnight at 110 °C. The yields of the synthesized carbons were expressed as the amount of the product formed per gram of the metal used (i.e., gCNFs/gNi). The carbon fibers obtained over unsupported Ni were denoted as CNF1, whereas the sample obtained over 10%Ni/CaCO_3_ was denoted as CNF2.

### Functionalization of carbon samples

The obtained CNFs were modified in order to introduce acid functional groups on their surface. The sample functionalization was performed with concentrated sulfuric acid or 4-benzenediazonium sulfonate generated in situ.

Modification with H_2_SO_4_ was carried out in a three-neck flask using 3 g of CNFs and 77 cm^3^ of concentrated sulfuric acid (pure p.a., 96%, Stanlab, Poland). The modification was performed at 140 °C for 20 h under an argon atmosphere (flow rate of 30 cm^3^/min) upon continuous stirring. The resulting material was filtered, washed with distilled water until a neutral pH of the filtrate was achieved, and then dried at 110 °C overnight. The final samples obtained in that way were denoted as CNF1_H_2_SO_4_ and CNF2_H_2_SO_4_.

Functionalization with diazonium salt was performed in a three-neck round-bottom flask immersed in a water bath and equipped with a magnetic stirrer and a thermocouple. 224 cm^3^ of distilled water and 4.5 g of CNFs were placed in the reactor. Then, 6.4 g of sulfanilic acid (pure p.a., Merck, Germany) and 2.6 g of sodium nitrite (pure p.a., Chempur, Poland) were added to the mixture. Subsequently, 35–38% hydrochloric acid (45 cm^3^; pure p.a., Chempur, Poland) was added dropwise. The modification was carried out at room temperature for 20 h. The resulting carbon products were filtered and washed with hot distilled water, followed by rinsing with methanol (99.8%, pure p.a., Avantor Performance Materials, Poland), DMF (pure p.a., Eurochem, Poland), and acetone (pure p.a., Stanlab, Poland). Finally, the samples were dried at 110 °C overnight. The obtained materials were denoted as CNF1_BDS and CNF2_BDS.

### Characterization of samples

In order to examine the porous structure of the prepared samples, a Quantachrome Autosorb IQ apparatus working at -196 °C with nitrogen as an adsorbate was used. Specific BET surface areas (S_BET_) of CNFs were calculated using the Brunauer–Emmett–Teller equation. On the other hand, the volumes of micropores (V_micro_) and the external surface areas (mesopores + macropores; S_ext_) of samples were determined applying the t-plot method. The total pore volumes (V_tot_) of the materials were calculated from the amount of N_2_ adsorbed at a relative pressure close to unity. The quantitative elemental analysis was performed with a Vario EL III EA apparatus. The morphological features of the carbon nanofibers were analyzed using electron microscopy. A JEOL JEM-1200 II apparatus was used for TEM (transmission electron microscopy) investigations, whereas a Helios NanoLab 660 Dual Beam apparatus was applied for XHR-SEM (extreme high resolution scanning electron microscopy) measurements. Thermogravimetric (TG) analysis was done on a Setaram Setsys 1200 thermal analyzer under N_2_ or air atmosphere, using 10–15 mg of a sample. The test temperature range between 20 and 1000 °C and a heating rate of 10 °C/min were applied in all tests. The TG and DTG curves were prepared. Powder XRD patterns were obtained with a powder diffractometer Bruker D8 Advance equipped with a Johansson monochromator (λCu Kα1 = 15,406 Å) and a silicon strip detector LynxEye.

### Catalytic tests

Esterification of glycerol with acetic acid was carried out in a three-neck round-bottom flask (50 cm^3^), equipped with a magnetic stirrer, reflux condenser and a thermocouple. 28.9 cm^3^ of acetic acid (99.0–99.5%, pure p.a., Stanlab, Poland) and 0.7 g of catalyst were put into the reaction system. The obtained mixture was heated to 80 °C under nitrogen flow. After reaching the desired temperature, glycerol (99.5%, pure p.a., Stanlab, Poland) was introduced into the flask. The reaction was carried out with a glycerol to acetic acid molar ratio of 1:3 or 1:6. In order to monitor the progress of the reaction, aliquots of the reaction mixture were taken for analysis after different time intervals. The analysis of samples was performed using a gas chromatograph equipped with a capillary column InterCap WAX (length of 30 m, internal diameter of 0.53 mm, film thickness of 1.0 µm) and a FID detector, at the temperature between 130 and 230 °C. The catalytic performances of samples were expressed as conversion of glycerol and selectivity to various esters. A blank test (without a catalyst) was performed under similar conditions. For comparison purposes, Amberlyst 15 was also examined. The repeatability of all tests was very high, and the calculated standard deviations were generally lower than 2% for glycerol conversions and lower than 1% for selectivities and yields of acetins.

## Results and discussion

Carbon materials prepared in this study were characterized using various techniques, namely scanning and transmission electron microscopy (SEM and TEM, respectively), thermogravimetric (TG) analysis, X-ray diffraction technique (XRD) as well as elemental and textural analyses. Furthermore, the carbon samples were used in the reaction of glycerol with acetic acid to produce acetins. The obtained results and their discussion are presented in the sections below.

### Characterization of the carbon samples

**Figure 1:**
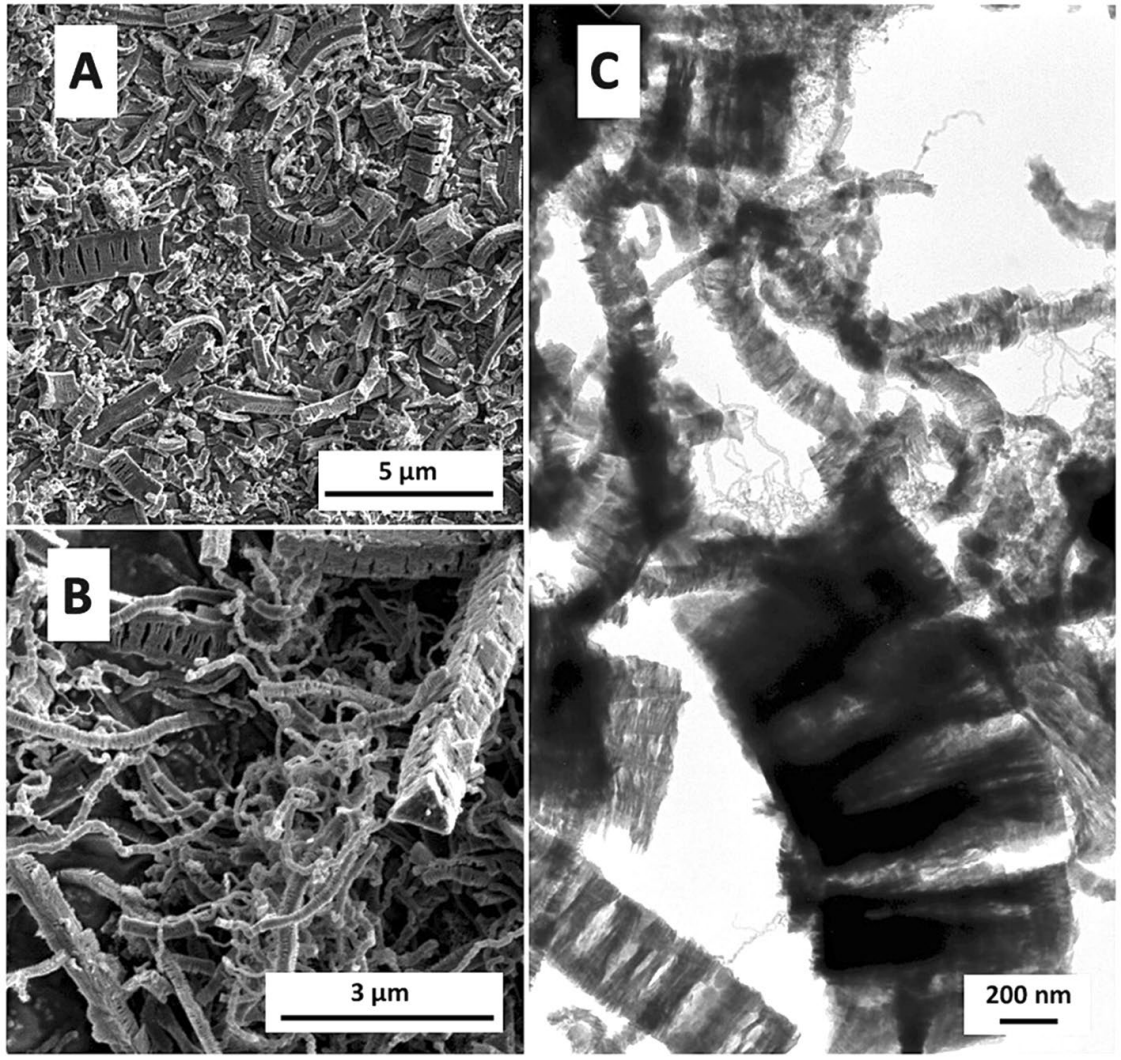
Typical XHR-SEM (**A**, **B**) and TEM (**C**) images of the CNF1 sample.

Figure 1A–C shows the morphology of carbon structures obtained in the presence of Ni used as a growth catalyst when a mixture of C_2_H_4_ and H_2_ was applied (i.e., the CNF1 sample). Coiled CNFs of small diameters and circular cross-sections were formed in this case; however, the final product also contained a lot of fibers with square or triangular cross-sections and rough, slotted surfaces (Fig. 1B). Analysis of Fig. 1C clearly suggests that CNFs have a platelet structure with the graphene sheets arranged perpendicular to the fiber axis. In the same image, also carbon nanotubes of small diameters can be noticed.

**Figure 2:**
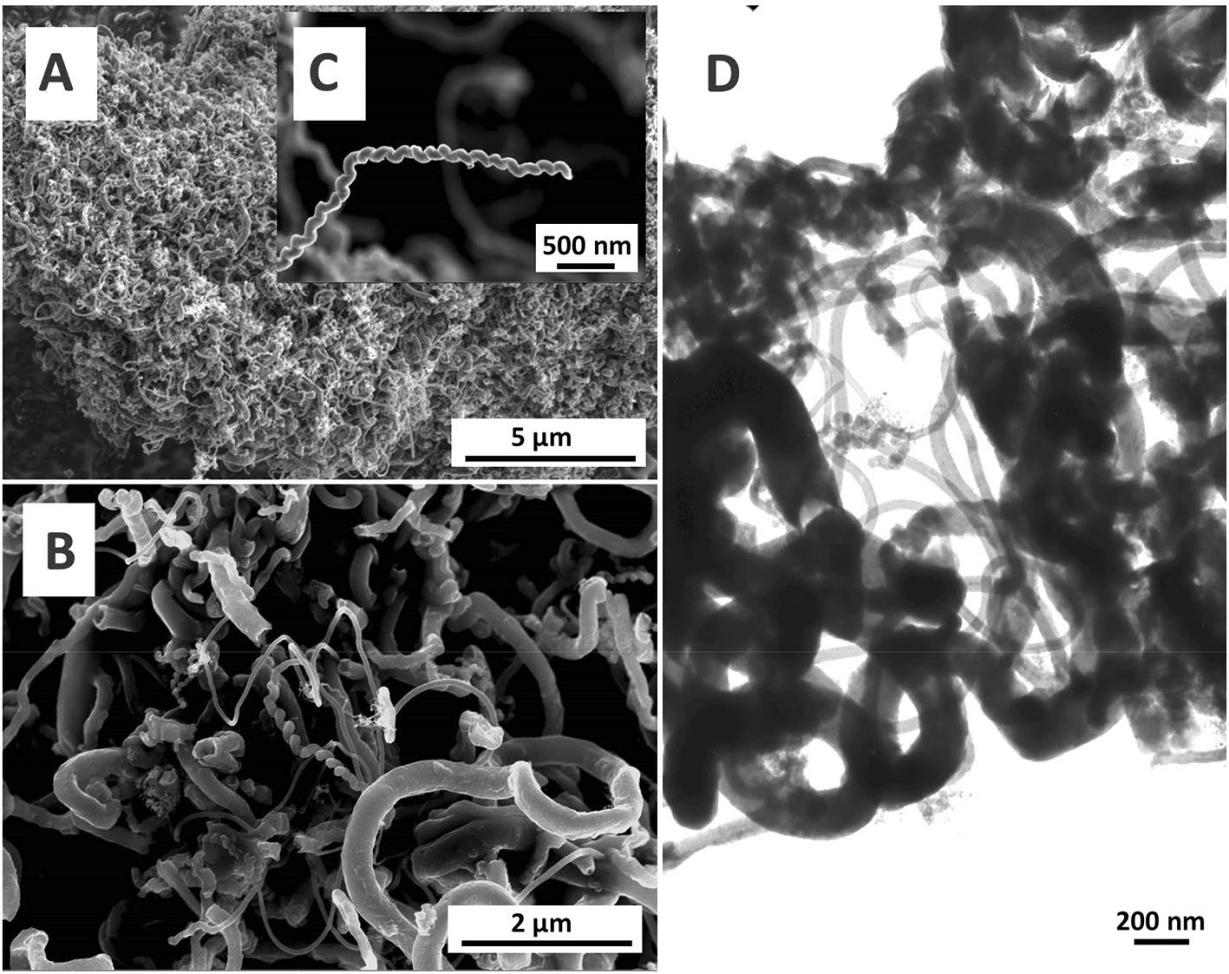
XHR-SEM (**A**, **B**, **C**) and TEM (**D**) images of the CNF2 sample.

When supported Ni and pure ethylene were used, the carbon deposit formed (i.e., the CNF2 sample) was different from that collected from pyrolysis of a 25%C_2_H_4_/75%H_2_ mixture (i.e., CNF1). As can be seen in Fig. 2A and B, the prepared CNF2 material was rather heterogeneous in nature. It consisted of a dense network of tangled fibers of various lengths and diameters. Larger-size fibers (diameter up to about 300 nm) predominated; however, long fibers of small diameters (of about 30 nm) were also present. Interestingly, part of the product obtained was in the form of helical carbon fibers, as can be seen in the XHR-SEM pictures presented in Fig. 2B and C. The surfaces of all the structures were rather smooth. This was corroborated by the XHR-SEM image shown in Fig. 2B. Furthermore, TEM picture presented in Fig. 2D confirms that the carbon structures formed were rather in the form of fibers than tubes. It is worth emphasizing that the yields of both types of materials were significant and equal to 143 and 130 gCNF/gNi for CNF1 and CNF2, respectively.

TG analysis under air atmosphere can be a very informative tool used in the characterization of carbon nanotubes and nanofibers (CNTs and CNFs, respectively), especially describing their quality and purity. This is mainly due to the fact that different structural forms of carbon exhibit different reactivity towards oxidation. In general, amorphous carbon is suggested to be less resistant to oxidation than its graphitic form and typically burns out at the temperature below 400 °C^32–34^. Purified CNFs and CNTs are more stable and are oxidized between 450 and 650 °C^32,34,35^. The onset of the oxidation temperature measured for these types of filaments depends on the number of defects, among others, e.g., less defective structures are oxidized at higher temperatures than highly-defective ones^34^.

**Figure 3:**
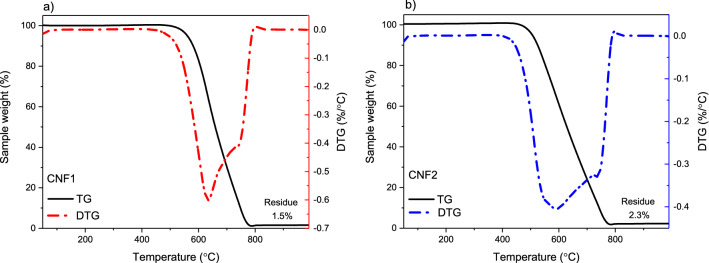
TG and DTG curves of CNF1 (**a**) and CNF2 (**b**) samples (air atmosphere).

**Figure 4:**
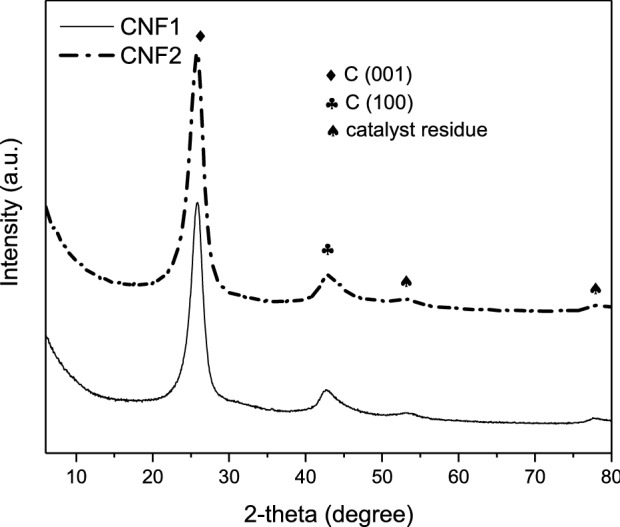
The XRD patterns of the carbon samples obtained from ethylene via the CCVD process.

Figure 3 shows the results of thermogravimetric analysis (performed in an air atmosphere) of the obtained carbon products. As can be seen from the TG curves (black lines in the graphs), both samples were stable up to about 500 °C. The oxidation was initiated above this temperature and a rapid decrease in the sample weight was reported in both cases. Finally, almost complete weight loss was observed at the temperature of about 780–800 °C, which was due to combustion of all the carbon substance. The content of residue after complete oxidation was very low (between 1.5 and 2.3%), which proves the efficiency of the purification step (see “Experimental part” section). Furthermore, it should be stressed that the residual mass contained the oxide of metal catalyst formed under the conditions of TG analysis (air atmosphere) and not the equivalent of pure metal taken for the CCVD reaction^36^. Thus, the contamination of CNF1 and CNF2 samples with the metal catalyst was even lower. The same was reported for a selected series of modified carbon fibers. The effectiveness of purification step was also confirmed by the XRD profiles of CNF1 and CNF2 (Fig. 4), showing barely marked reflections belonging to species other than carbon, most likely to Ni crystallites (on the basis of XRD pattern presented elsewhere, the presence of CaCO_3_ can be excluded^31^), which is also in line with the results of TG analysis performed in an air atmosphere. Only DTG signals at a high-temperature range (Fig. 3) were reported for the tested samples (with the minima of the major peaks at about 590–620 °C), indicating the presence of ordered filamentous carbon. The lack of low-temperature events in the DTG profiles can suggest that both examined samples were quite pure and did not contain significant amounts of amorphous carbon. This is also in line with the XRD results of the CNF1 and CNF2 samples (Fig. 4), showing the dominant contribution of an intense, narrow (002) diffraction peak at 2-theta of about 26° belonging to graphite^37,38^, and indicating a high degree of graphitization and a well-ordered crystallographic arrangement of the obtained CNFs. Interestingly, the shape of the DTG signals presented in Fig. 3a and b suggests the presence of two peaks, reflecting different stability of the obtained carbon structures towards oxidation, with minima at about 620 and 780 °C for CNF1 and 590 and 750 °C for CNF2. In both cases, the former can be attributed to more reactive phase, showing higher disorder degree, while the latter can be ascribed to less reactive, more organized carbon^39^. The shapes of the DTG profiles can also be related to differences in diameters of the formed filamentous carbon^36,40^.

**Figure 5:**
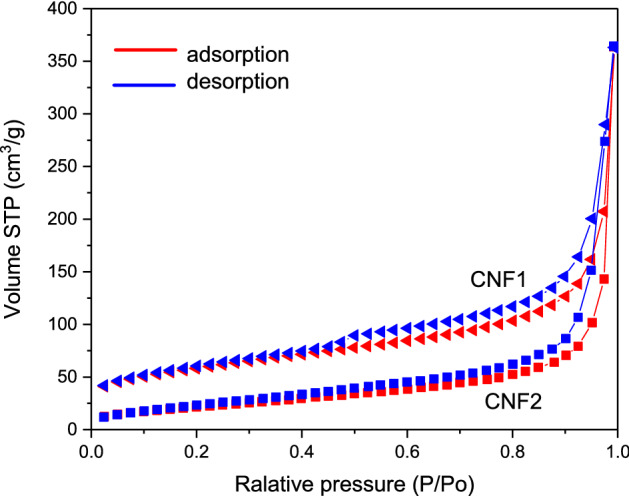
Nitrogen adsorption–desorption isotherms obtained for CNF 1 and CNF2.

Table 2 presents porous features of the obtained CNFs. As can be seen, the initial samples showed relatively high surface areas, which were 200 and 82 m^2^/g for CNF1 and CNF2, respectively. The CNF2 carbon contained almost only meso- and macropores as V_micro_ ≈ 0. In contrast to CNF2, CNF1 also had some content of micropores, as V_micro_ for this sample was 0.05 cm^3^/g, i.e., about 9% of the total volume of pores (V_tot_), and S_ext_ (i.e., surface area of meso- and macropores) was about 55% (thus, the area of micropores was about 45%). This result is in line with the SEM/TEM findings and it is probably related to the presence of slits and voids in the platelet structure of CNF1 (Fig. 1). The presence of mesopores in the structure of both samples was also confirmed by the shape of N_2_ adsorption/desorption isotherms of these carbons, with a hysteresis loop typical for mesoporous materials (Fig. 5).

**Table 2:** Analysis of the porous structure of samples.

Sample	S_BET_ [m^2^/g]	S_ext_ [m^2^/g]	V_tot_ [cm^3^/g]	V_micro_ [cm^3^/g]
CNF1	200	109	0.56	0.05
CNF2	82	77	0.56	< 0.01

The results of elemental analysis of the obtained materials (before and after functionalization) are presented in Table 3. The data indicate that all the prepared samples showed a very high content of carbon, which was between 92.8 and 97.4% (higher contents were observed for the unmodified samples). Most importantly, the selected functionalization methods led to successful incorporation of S to CNFs (the S content in the modified samples between 0.3 and 1.0%). However, the efficiency of these modifications was considerably lower than that observed for other carbon materials, such as carbon xerogels and spheres (for which the S contents were between 1.4 and 4.3%)^17^, ordered mesoporous carbons (S = 1.4% and 7.0%)^18^, or activated carbons (the S content of 1.5–2.8%)^41^. Significantly higher amounts of sulfur were introduced to the virgin CNFs during functionalization with diazonium salt (BDS-modified CNFs contained 2–3 times more S than H_2_SO_4_-modified carbons). The highest amount of S (1.0%) was observed in the case of CNF1_BDS, and this quantity was about 1.6 times higher than that measured for CNF2_BDS. The direct cause of this phenomenon might be differences in the morphology between CNF1 and CNF2 that affected the samples’ susceptibility to modification. As shown in Table 2, CNF1 had higher surface area. Moreover, in contrast to CNF2, the surface of CNF1 fibers was not smooth but presented a platelet structure with active graphene sheet edges (compare Figs. 1 and 2).

**Table 3:** Chemical composition of the prepared CNF samples (in wt.%, dry basis) and the concentration of surface –SO_3_H groups (in mmol/g).

Sample	C	H	N	S	O^a^	–SO_3_H^b^
CNF1	97.4	0.2	0.1	0.0	2.3	0.00
CNF2	97.0	0.2	0.1	0.0	2.7	0.00
CNF1_BDS	92.8	0.4	0.4	1.0	5.5	0.31
CNF1_H_2_SO_4_	94.6	0.2	0.1	0.3	4.8	0.09
CNF2_BDS	95.1	0.3	0.3	0.6	3.7	0.19
CNF2_H_2_SO_4_	96.0	0.2	0.1	0.3	3.5	0.09

^a^Calculated by difference.

^b^Calculated from the content of sulfur.

Both types of parent CNFs contained only a small amount of oxygen (up to 2.7%), which in general increased significantly after the modifications. The rise was more prominent in the case of CNF1, for which the oxygen content increased from 2.3 to 4.8% for the sample modified with H_2_SO_4_ and to 5.5% for the carbon fibers modified with BDS. Overall, the discussed increase in the sulfur and oxygen contents can indicate the formation of –SO_3_H groups on the surface of modified samples. In the case of CNFs modified with H_2_SO_4_, the formation of different oxygen functionalities is also possible due to the oxidative nature of sulfuric acid^42^.

**Figure 6:**
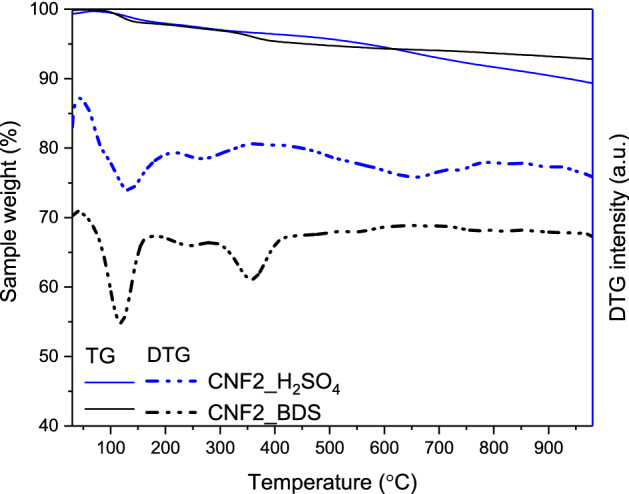
Thermal analysis of the modified carbon nanofibers under N_2_ atmosphere.

Figure 6 presents the results of TG analysis of selected modified samples performed in an inert atmosphere. As can be observed from the TG patterns, both tested materials showed some weight loses when temperature increased. Finally, the sample weight was reduced by about 10.9% in the case of CNF2_H_2_SO_4_ and by 7.3% in the case of CNF2_BDS. According to the DTG profiles of CNFs, different processes were responsible for these decreases. First, both DTG curves show a peak with a minimum at the temperature of about 120 °C, which probably corresponds to the release of physically adsorbed water. Further, CNF2_H_2_SO_4_ has a weak signal with a minimum at about 250 °C, which is probably due to the decomposition of sulfonic groups^42,43^. In turn, quite a significant weight loss reported above 500 °C for this sample is most likely related to the decomposition of surface oxygen groups that can also be created during the reaction of carbons with concentrated sulfuric acid^42,43^.

Interestingly, the DTG profile of CNF2_BDS shows an intense peak with a minimum at the temperature of 360 °C, ascribed in the literature to –PhSO_3_H groups^44^, suggesting higher thermal stability and a higher degree of functionalization with sulfur of the CNF2_BDS sample than CNF2_H_2_SO_4_. Indeed, higher amounts of sulfur were measured in CNF2-BDS (see EA results in Table 3). Importantly, the absence of signals at the temperatures above 500 °C in the DTG profile of the sample modified with BDS can suggest that all the oxygen introduced during the sample modification is present in the form of –SO_3_H structures (in contrast to CNF2_H_2_SO_4_).

### Catalytic activity of the prepared carbon fibers

Acetylation of glycerol (Gly) with acetic acid (AA) proceeds in three consecutive reversible reactions, producing monoacetins (MA), diacetins (DA), and triacetin (TA) successively and water as a by-product. The stoichiometric reaction requires 1 mol of Gly and 3 mol of AA to give one mol of the most desired acetin, i.e., TA^45,46^. The reaction steps are listed below:1

**Figure 7:**
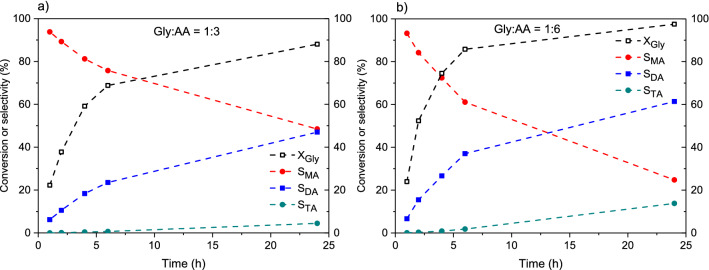
Catalytic performance of CF1_BDS sample in glycerol esterification using glycerol (Gly) to acetic acid (AA) molar ratios of 1:3 (**a**) and 1:6 (**b**); X_Gly_—conversion of glycerol, S_MA_, S_DA_ and S_TA_—selectivity to mono-, di- and triacetins, respectively.

Shifting the equilibrium of the above-mentioned processes towards acetins is typically performed by using an excess of acetic acid or by removing water from the reaction mixture^45^. In order to improve the process efficiency, the former approach was used in the current study and the glycerol esterification was carried out with the increased amount of AA (Gly/AA molar ratio of 1:6). For the sake of comparison, the reaction under stoichiometric conditions (1:3) was also conducted. The results of catalytic performance of a selected catalyst (CNF1_BDS) are presented in Fig. 7. As can be seen in the graphs, at the first time point measured (i.e., after 1 h), similar conversions of glycerol (X_Gly_) and selectivities to the particular products (i.e., S_MA_, S_DA_ and S_TA_) were noted when using glycerol to acetic acid molar ratios of 1:3 and 1:6. However, with extended reaction times, significant differences appeared in the results obtained at different molar ratios of the reactants. For instance, the glycerol conversion measured after 6 h in the reaction performed at the Gly/AA molar ratio of 1:3 was about 70%, whereas in the process performed with the increased amount of acid (i.e., Gly/AA molar ratio of 1:6), it was almost 90%. Importantly, change in the Gly/AA molar ratio from 1:3 to 1:6 resulted in significantly improved selectivities to higher acetins (i.e., DA and TA). Thus, the mixture of products obtained after 24 h in the process performed with the lower amounts of acetic acid still contained about 48% of monoacetins with only traces of triacetin, whereas in the reaction conducted at 1:6 Gly/AA molar ratio, the combined selectivity to higher esters (S_DA+TA_) was about 76%. Finally, the Gly/AA molar ratio of 1:6 was selected for the remaining experiments over CNFs, as these conditions were found to present a good compromise between the obtained catalytic results and the consumption of reagents. Applying higher amounts of acetic acid was not tested for economic reasons. Furthermore, our previous studies showed that using a mixture of reagents at a higher molar ratio of AA to Gly (such as 9:1) is not profitable and does not give particularly good outcomes^18^.

**Figure 8:**
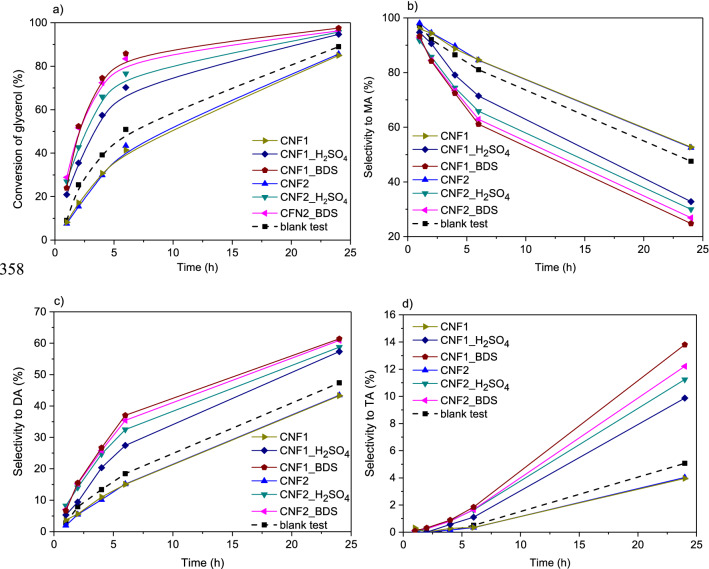
Conversion of glycerol (**a**) and selectivities to acetins (**b**, **c**, **d**) obtained in glycerol acetylation performed with a glycerol to acetic acid molar ratio of 1:6 over the prepared carbon fibers.

Figure 8 presents the catalytic performance of the CNFs when using Gly/AA molar ratio of 1:6. In order to assess the real activity of the carbons, the results obtained in the reaction performed without a catalyst (i.e., blank test) were also shown. As can be seen, under the applied reaction conditions, MA, DA and TA were produced even in the absence of a catalyst. However, in the blank test the conversion of glycerol was not high (about 50% after 6 h), and the selectivity was limited mainly towards MA or the mixture of MA and DA, which were both found in similar amounts after 24 h, instead of the desired higher concentrations of DA and TA. The results of the blank experiment show that glycerol can be quite easily transformed to MA and DA (mainly 1,3-disubstituted DA), but it is difficult to convert intermediates to the trisubstituted product due to the steric hindrance, as it was shown earlier^47^. The use of modified CNF catalysts increased the glycerol conversion (at each stage of the process compared to the blank test, see Fig. 8a) and enhanced the combined selectivity to DA and TA (Fig. 8c and d).

When considering the effect of reaction time, the conversion of glycerol increased significantly over time, especially when modified samples were used. In this case, a rapid glycerol consumption was reported in the first few hours. The high values of X_Gly_ were obtained after 24 h of the reaction, however, for the best catalysts quite satisfactory results were achieved just after 6 h of the process (Fig. 8a). Initially, monoacetins were mainly produced in the esterification (Fig. 8b); however, the amounts of these products decreased significantly over time, as MAs were converted to higher esters. Consequently, selectivities to DA and TA increased with the decrease in the selectivity to MA (Fig. 8c and d). The observed phenomenon is consistent with the previous reports and confirms the consecutive reaction mechanism (see Eq. (1)) ^7^. Finally, for the best sample, a high combined selectivity to DA and TA was observed with only limited selectivity to MA. This is an important finding because DA and TA are the products of interest, which, additionally, do not require separation before application in biodiesel and petro fuel^48^.

**Figure 9:**
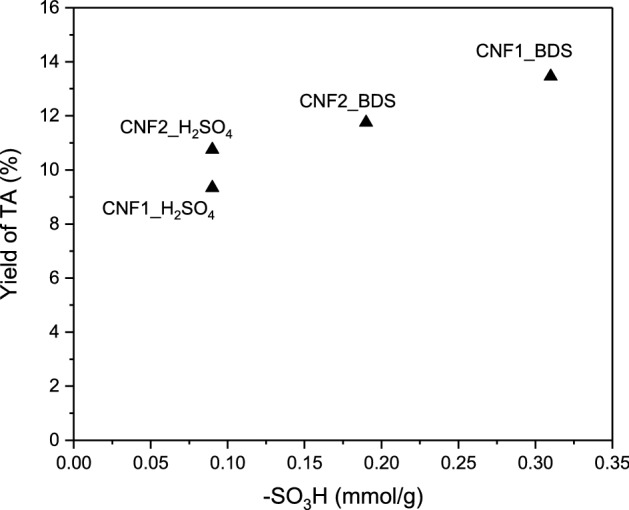
The yields of triacetin (TA) obtained using modified CNFs produced from ethylene versus the content of –SO_3_H groups on the sample surfaces.

It is commonly known that glycerol acetylation is a classical acid-catalyzed reaction that is strongly dependent on the strength and the amount of acidic sites^49^. Thus, to endow the surface of the prepared CNFs with strongly acidic nature, our samples were modified with H_2_SO_4_ or BDS to functionalize them with –SO_3_H groups of strong Brönsted acidity (pKa of 0.7^42^). The applied modifications resulted in the increase in the S content (which was discussed earlier, see EA in Table 3) and in the introduction of sulfonic groups (as all the sulfur was incorporated into the carbon matrix in the form of –SO_3_H groups, as shown before^18,41^). The applied functionalizations drastically improved the catalytic performances of CNFs (Fig. 8). Based on the catalytic results and the data presented in Table 3, it can be concluded that the catalytic activities of carbons were closely related to the contents of sulfonic groups in the samples. Thus, the best results were recorded in the esterification carried out in the presence of CNFs modified with BDS that introduced significantly higher amounts of S into the carbons compared to sulfuric acid. CNF1_BDS, for which the highest density of –SO_3_H was calculated (Table 3), caused almost complete conversion of glycerol in 24 h, producing a mixture containing about 62% of DA and about 14% of TA after 24 h. On the other hand, the performance of CNFs modified with sulfuric acid (with a lower number of sulfonic groups; Table 3) was far worse. Finally, the analysis of the attained data allowed us to conclude that there is a relationship between the –SO_3_H content in the tested carbon nanofibers (Table 3) and the yield of the most desirable reaction products. Figure 9 presents the dependence between TA yields and the concentrations of sulfonic groups in CNFs. As can be seen there is a clear correlation between these two parameters, namely, the higher the –SO_3_H content, the higher the yield of TA. This simply means that –SO_3_H groups are essential for transformation of glycerol into valuable products, as they facilitate the formation of less thermodynamically favored (due to the steric hindrance) triacetin. It should be stressed, however, that it is difficult to predict the extent to which the discovered relationship between the content of sulfonic groups and TA yields would be applicable, as the study included samples where the number of –SO_3_H groups was within a narrow range. Furthermore, it cannot be excluded that also other parameters such as the sample morphology and the possible presence of oxygen groups influence the catalytic performances of the prepared samples. Future studies should address these issues.

**Figure 10:**
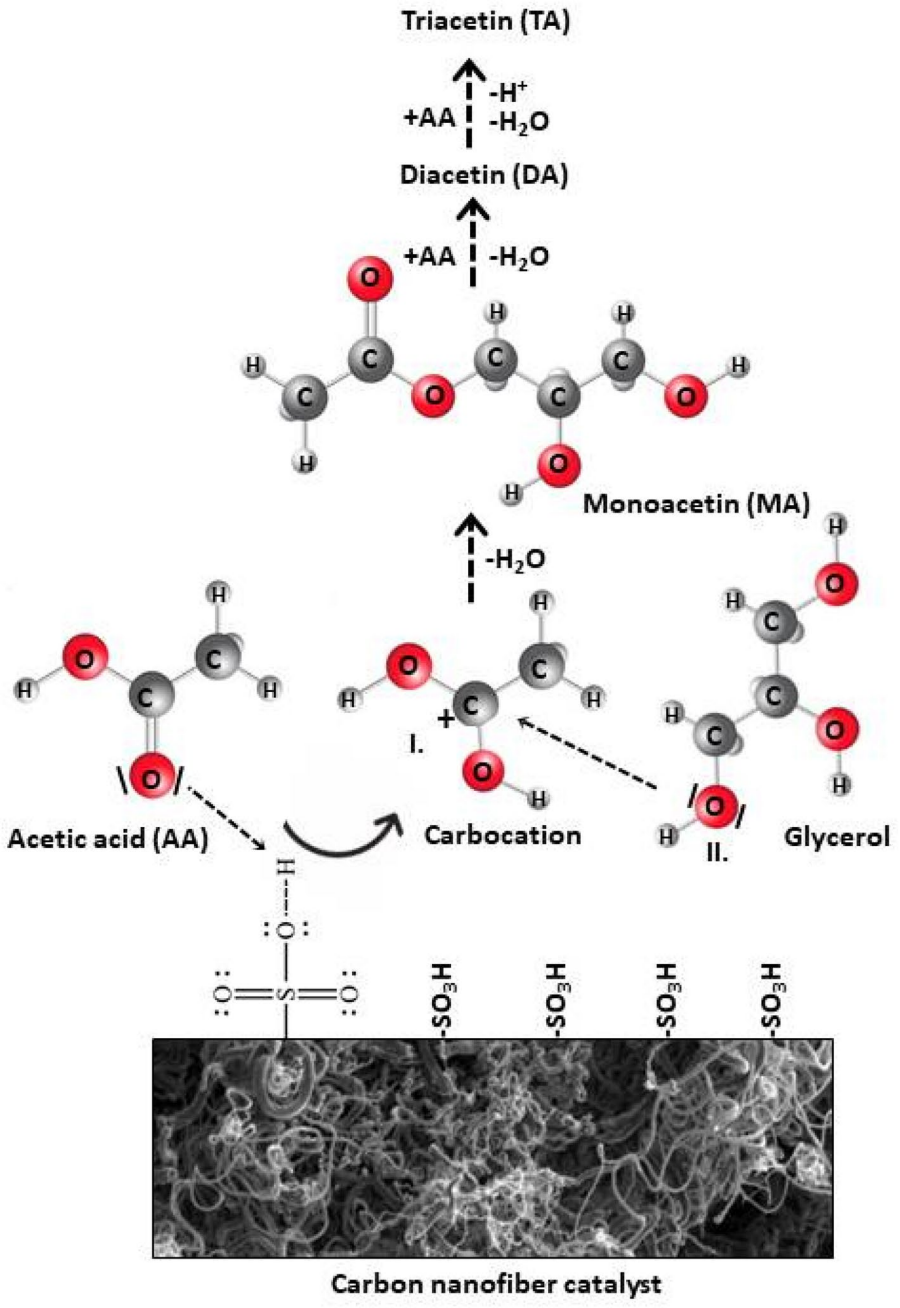
A simplified mechanism of glycerol esterification with acetic acid over SO_3_H-bearing CNFs.

It is commonly believed that glycerol acetylation with acetic acid follows the Fischer esterification mechanism^7,46^. Thus, the acetylation using CNF-SO_3_H catalysts is a reaction involving Brønsted acid –SO_3_H groups (Fig. 10). Initially, a proton from the acid catalyst is used for protonation of oxygen from a carbonyl group of acetic acid (forming carbocation I). In the second step, the oxygen atom from one of the primary hydroxyl groups (II) of glycerol acts as a nucleophile and attaches to the sp^2^ carbon formed in the first step. This also eliminates the proton from II. A series of fast equilibrium proton exchanges taking place in –OH groups of acetic acid results in the formation of a new ester bond between the carboxyl group carbon and the oxygen in glycerol and simultaneous elimination of a molecule of water. This process is repeated with the remaining –OH groups of a glycerol molecule and AA, forming finally triacetin and releasing H^+^. From this mechanism, it is obvious why the yield of triacetin increases with increasing amount of sulfonic groups, as presented in Fig. 9.

**Figure 11:**
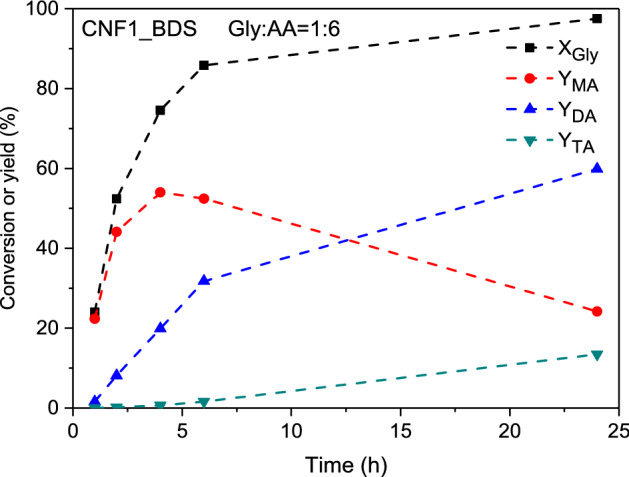
The catalytic performance of CNF1_BDS over time expressed as the conversion of glycerol and yields of individual acetins; X_Gly_—conversion of glycerol, Y_MA_, Y_DA_ and Y_TA_—yield of mono-, di- and triacetins, respectively.

Figure 11 depicts the catalytic performance of CNF1_BDS (the best sample) over time expressed as yields of individual products.

**Figure 12:**
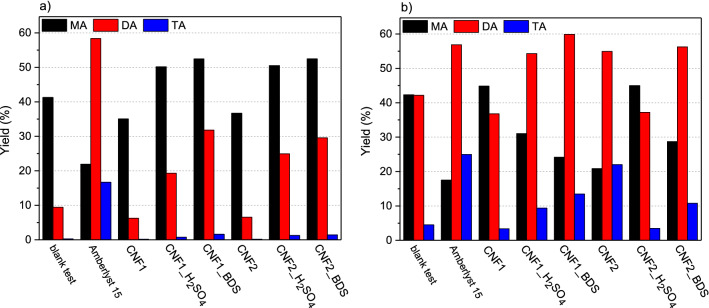
Yields of acetins (MA, DA and TA—mono-, di- and triacetins, respectively) obtained in the esterification performed at a Gly/AA molar ratio of 1:6 after 6 h (**a**) and 24 h (**b**).

As can be seen, the best results were attained after 24 h; however, quite reasonable outcomes could also be obtained after 6 h of the process. Thus, Fig. 12 compares the results of acetin yields achieved in the esterification performed in the prepared CNFs as well as a commercial catalyst (Amberlyst 15) after 6 and 24 h. As can be observed from these graphs, after 6 h of the reaction, the modified carbons produced higher yields of acetins compared to the unmodified samples and the blank test; with the main products formed in these cases being monoacetins. The best catalytic performance was shown by CNF1_BDS, presenting the highest number of –SO_3_H sites, for which the yield of DA + TA was about 34% and the MA yield was of about 52%. However, better catalytic performance was shown by Amberlyst 15. The progress in the reaction time caused significant changes in the obtained results, as after 24 h the distributions of acetins were considerably altered. In the case of functionalized CNFs, after 24 h DA dominated and the amount of TA formed was significantly raised. The highest combined yield of DA and TA, i.e., the most desired products, was attained again for CNF1_BDS (about 73%), showing that this sample was the most active catalyst among all the carbon samples tested in this work. Amberlyst 15 worked more effectively again; however, the differences between the performance of Amberlyst 15 and that of the prepared carbons were smaller than those obtained after 6 h of the reaction.

The main limitation of the study is the inability to easily compare the obtained results to those previously published. This is due to the varied experimental conditions used by different research groups, namely reaction temperature, glycerol to acetic acid molar ratio, catalyst concentration, or type of a reactor used (see also Introduction and Table 1). A direct comparison of the effectiveness of catalysts would require expressing their performances as activities or TOF/TON numbers. Meanwhile, this is a rather rare practice in the case of glycerol acetylation, especially over carbon materials. Nevertheless, the catalytic performance of the CNFs developed in the present study was compared to that of various carbons prepared by our group in the past and tested under the same acetylation conditions. Finally, in order to achieve carbon neutral or net zero carbon industry targets, a lifetime impact of the proposed CNFs catalysts on the environment should be analyzed applying for example LCA analysis^50–52^.

Table 4 presents a comparison of the catalytic performance of the CNFs developed in the present study with that of different carbon catalysts reported by us previously. As can be observed, the worst catalytic results in the process were obtained using carbonized hydrothermal carbon modified with BDS (HTC500-BDS), which was ascribed to inefficient functionalization of the starting carbon material with diazonium salt. Significantly better performance was shown by modified carbon xerogels, ordered mesoporous carbon, and carbon spheres, which produced a mixture containing mainly DA and TA at the temperature of 80 °C. Carbon obtained by partial carbonization (i.e., C_starch) worked even more effectively, as it showed high activity towards formation of DA and TA, giving high glycerol conversion and selectivities to di- and triacetins just within 2 h of the reaction performed at 110 °C. The carbon fibers acquired in this work allowed us to obtained quite reasonable results (i.e., high combined selectivity to DA and TA) at relatively low temperature; however, this required quite a long reaction time. The worse results attained with modified CNFs compared to those obtained with the other tested carbon-based samples are probably due to the low degree of CNFs’ functionalization (see Table 3).

**Table 4:** Comparison of the catalytic performance of the CNFs developed in the present study with that of different carbon catalysts reported by us previously.

Sample	Gly/AA molar ratio	Temp. (°C)	Time (h)	Selectivity (%)			Glycerol conversion (%)	References
				MA	DA	TA		
CX-H_2_SO_4_	1:6	80	6	40	~ 54	6	~ 94	^17^
C_SBA-15_-BDS	1:6	80	6	29	61	10	~ 96	^18^
HTC-H_2_SO_4_	1:6	80	6	28	60	12	~ 95	^19^
HTC500-BDS	1:6	80	24	59	38.5	~ 2.5	82	^19^
C_starch	1:6	110	2	26	56	18	96	^20^
CNF1_BDS	1:6	80	24	22.5	61.5	~ 14	~ 98	This work

*Gly* glycerol, *AA* acetic acid, *MA* monoacetins, *DA* diacetins, *TA* triacetin, *CX* carbon xerogels, *C*_*SBA-15*_ ordered mesoporous carbon obtained by a hard template method, *HTC* hydrothermal carbon spheres, *HTC500* hydrothermal carbon spheres thermally treated at 500 °C, *BDS* 4-benzenediazonium sulfonate, *C_starch* carbon obtained from starch by partial carbonization, *CNF1* carbon nanofibers obtained from ethylene.

Overall, a direct effect of our study is the advancement of the existing knowledge on the catalysis on carbons, leading to a new understanding of this topic, and thus to future innovations based on metal-free catalytic systems. This is extremely important when taking into account the fact that metals, especially some transition metals, are rare, expensive, toxic, and environmentally harmful, which makes their usage non-sustainable and contrary to the main goals of the United Nations (namely, goal #12) of sustainable consumption and production patterns^53^. Furthermore, our research not only determined the suitability of CNFs in the glycerol acetylation process, but also established the relationship between the yields of acetins formed and the CNF surface structure. These findings set the direction for further studies in the field, which should focus on the preparation of samples with abundant and strong surface acidic groups such as –SO_3_H, using various methods^42,54^.

## Conclusions and future directions

Carbon nanofibers (CNF) were prepared with high yields using ethylene as a carbon source and Ni-type catalysts. The CNF samples were modified with sulfuric acid or 4‐benzenediazonium sulfonate (BDS) prepared in situ. The results of elemental analysis confirmed introducing functional groups containing sulfur and oxygen on the CNF surface. Modification of carbon nanofibers with BDS resulted in the introduction of higher amounts of S onto the surface of these materials compared to the modification with H_2_SO_4_, thus being a more efficient functionalization method. The obtained CNF materials were tested in the glycerol esterification process. The parent carbon samples worked inefficiently, and the displayed catalytic activity resulted from the autocatalytic nature of the reaction. The applied functionalizations significantly improved the catalytic performance of CNFs in the tested reaction, so the modified samples produced high yields of MA and DA just in 6 h. However, the more desired TA was formed in notable lower amounts. The reaction itself was carried out in two variants—with the use of a glycerol to acetic acid molar ratio of 1:3 and 1:6. The results obtained indicated that higher concentration of acid had a positive effect on the conversion of glycerol and selectivity to higher acetins. It was also found that there is a relationship between the –SO_3_H content on CNF surface and the yield of the most desirable reaction products formed in glycerol acetylation, i.e., the higher the –SO_3_H content, the higher yields of more substituted acetins (i.e., DA and TA).

In view of the above, the future work is recommended to synthesize structure- and size-controlled CNFs. These samples should be further functionalized to maximize the content of surface strongly acidic groups promoting the formation of higher acetins in glycerol acetylation (as established in this research). Therefore, various modification methods and method parameters should be analyzed. An important issue would also be a comparison of the activity of the produced carbon nanofibers (expressed as reaction rates or TOF/TON numbers) to that of other catalysts reported in literature in order to assess their real competitiveness against other catalytic systems. Future studies should also look into the sustainability features of the results obtained, using advanced sustainability assessment tools such as life cycle assessment (LCA) analysis. Finally, different flow reactor configurations and their application towards glycerol transformation should also be explored for the most promising samples.

## Abbreviations

CCVD: Catalytic chemical vapor deposition
TA: Triacetin
DA: Diacetin
MA: Monocetin
MTBE: Methyl tert-butyl ether
ETBE: Ethyl tert-butyl ether
DMF: Dimethylformamide
CNF1: Carbon fibers obtained with unsupported Ni + 25% C_2_H_4_/75%H_2_
CNF2: Carbon fibers obtained with 10%Ni/CaCO_3_ + 100% C_2_H_4_
TEM: Transmission electron microscopy
SEM: Scanning electron microscopy
TG: Thermogravimetric analysis
XRD: X-ray diffraction analysis
Gly: Glycerol
AA: Acetic acid
BDS: 4‐Benzenediazonium sulfonate
S_BET_: Apparent surface area
S_ext_: External surface area
V_tot_: Total pore volume
V_micro_: Micropore volume
